# Tropomyosin-based cross-reactivity and asymptomatic shellfish sensitization in patients with perennial allergy

**DOI:** 10.3389/falgy.2025.1598583

**Published:** 2025-06-27

**Authors:** Moritz Maximilian Hollstein, Marie Charlotte Schuppe, Katharina Klara Hahn, Prasad Dasari, Susann Forkel, Caroline Beutner, Timo Buhl

**Affiliations:** ^1^ Department of Dermatology, Venereology and Allergology, University Medical Centre Göttingen, Göttingen, Germany; ^2^ Lower Saxony Institute of Occupational Dermatology, University Medical Centre Göttingen, Göttingen, Germany

**Keywords:** tropomyosin, perennial allergy, food allergy, IgE cross-reactivity, shellfish

## Abstract

**Background:**

Mite, cockroach, and shellfish (crab, clam, and shrimp) proteins share allergenic epitopes. The determination of specific IgE (sIgE) against cockroach (*Blattella germanica,* relevant in asthma) and shellfish allergens (relevant in food allergy) using whole-body extracts necessitates detailed knowledge of IgE cross-reactivity.

**Objective:**

This study aimed to evaluate whether cross-reactivity between aeroallergens and food allergens is clinically relevant and whether subjects with mite and/or cockroach sensitization are at risk of false-positive results in shellfish food allergy diagnostics.

**Methods:**

In this cross-sectional, single-center study, we recruited 200 patients with elevated sIgE against ≥1 allergen at random from our outpatient clinic and assessed allergic comorbidity. We analyzed sIgE against mites (*Dermatophagoides pteronyssinus, Dermatophagoides farinae,* or *Blomia tropicalis*), German cockroach (*B. germanica*), crab, clam, and shrimp whole allergen extract, as well as sIgE against mite tropomyosin Der p 10 and shrimp tropomyosin Pen a 1 (in a subpopulation), using automated ImmunoCAP Specific IgE Tests.

**Results:**

During allergologic assessment, two participants reported previous anaphylaxis to fish and/or seafood and were excluded from further analysis. The final study population comprised 150 female and 48 male participants. Of these, 93 presented with positive sIgE against mites. As expected, participants with mite sensitization displayed an elevated prevalence of perennial asthma or allergic rhinitis (*p* < 0.001). Further, they were more often sensitized to German cockroach, crab, claw, or shrimp (each *p* < 0.001). Der p 10 and Pen a 1 sIgE levels were below the cutoff level (<0.35 kU/L) in all subjects. However, the correlation analyses revealed that tropomyosin sIgE explained between 24% and 55% of the variance (R^2^) in sIgE against clam, crab, German cockroach, or shrimp (each *p* < 0.001).

**Conclusion:**

Patients with mite sensitization have higher asymptomatic sIgE levels to shellfish. Even in patients with anti-tropomyosin sIgE levels below the cutoff level, anti-tropomyosin sIgE correlates strongly with sIgE against German cockroach, crab, clam, and shrimp. Our findings suggest large-scale false-positive results for sIgE to shellfish when analyzing patients with mite- or cockroach sensitization.

## Introduction

In allergic rhinitis, allergic asthma, and food allergy, organisms mount a type 2 immune response toward antigens from otherwise harmless exogenous substances, such as grass pollen or food protein. This immune response results in the production of antigen-specific IgE (sIgE) antibodies by plasma cells. The produced sIgE circulates through the organism and binds to mast cells. Upon re-exposition, this membrane-bound IgE can be crosslinked, triggering mast cell degranulation. Mast cell granules contain, amongst other mediators, histamine, a main mediator of allergic symptoms (1). Patient histories suggesting allergic rhinitis, allergic asthma, and food allergies are usually confirmed by measuring antigen-specific IgE in blood serum, by skin testing (2), or ultimately by allergen challenge (3). It is important to note that skin tests and sIgE measurements may only prove sensitization. The presence of clinical symptoms upon allergen exposure is a mandatory feature of allergic diseases (4). Therefore, sensitization does not automatically imply allergy. The reasons for this are not fully understood.

The prevalence of allergic sensitization, especially to airborne allergens, has increased significantly in recent decades (5–8). Among children in the Western world, asthma is the most prevalent chronic airway disease, affecting up to 14% of children aged 14 years and younger. A major risk factor for asthma is sensitization to cockroach antigens (9). Skin tests of US children with moderate-to-severe asthma found that 69% were sensitized to cockroach antigens (10). Allergen extracts have not yet been replaced by molecular diagnostics for mite and cockroach allergy diagnosis (11).

Food allergy is less common but is potentially lethal, as it may cause anaphylaxis. In the US and Germany, 10.8% (12) and 4.7% (13) of adults self-report an IgE-mediated food allergy, respectively. These numbers are likely far higher than the prevalence of IgE-mediated food anaphylaxis in the real world. For a layperson, IgE-mediated oral allergy syndrome (OAS) and intolerance reactions may easily be confused with a genuine food allergy that causes anaphylaxis. The proportion of Germans sensitized to foods (25.5%) is far higher than the above-mentioned prevalence of food allergy (7).

Among the most common foods causing anaphylaxis in adults are peanuts, tree nuts, and shellfish (14). In children, the most allergenic foods are milk, egg, peanut, tree nuts, fish, wheat, shellfish, and soy (15). The term seafood generally encompasses fish, crustaceans, and mollusks. The term shellfish is used more narrowly and refers to mollusks and crustaceans, but not fish (16). The major allergens in fish (parvalbumin) and shellfish (tropomyosin) do not overlap. Sensitization to shellfish is found in 6% of the US population (17), while self-reported shellfish allergy affects 2% (18).

In shellfish, i.e., mollusks and crustaceans such as shrimp, tropomyosin is considered to be the major allergen (19). Tropomyosin can be found in *Penaeus aztecus* (brown shrimp), *Crangon crangon* (North Sea shrimp), *Charybdis feriatus* (crucifix crab), *Macrobrachium rosenbergii* (giant freshwater shrimp), *Homarus americanus* (American lobster), and similar animals. However, it can also be found in *Dermatophagoides pteronyssinus* [house dust mite (HDM)], *Dermatophagoides farinae* (American house dust mite), *Blattella germanica* (German cockroach), and *Periplaneta americana* (American cockroach) (19).

This suggests IgE cross-reactivity between aeroallergens (cockroach and mites) and food allergens (shellfish) (20–22). This study aimed to evaluate whether this cross-reactivity is clinically relevant and whether subjects with mite and/or cockroach sensitization are at risk of false-positive results in shellfish food allergy diagnostics.

## Materials and methods

In this cross-sectional, single-center study, 200 patients with elevated sIgE against ≥1 allergen from our outpatient clinic were recruited at random and their allergic comorbidity was assessed. The participants’ sera were analyzed for sIgE against mites (*D. pteronyssinus, D. farinae,* or *B. tropicalis*), German cockroach (*B. germanica*), crab, clam, and shrimp whole extract, as well as sIgE against tropomyosin Der p 10 (tropomyosin from HDM), using automated ImmunoCAP Specific IgE Tests (Thermo Fisher, Waltham, MA, USA) following the manufacturer's instructions. In a subpopulation of subjects with suspected tropomyosin-based cross-reactivity (i.e., those with elevated levels of sIgE against any mite and crab, against any mite and German cockroach, or against German cockroach and crab) (*n* = 30), sIgE against Pen a 1 (tropomyosin from shrimp) was also evaluated. The level of sIgE antibodies was measured in kU/L. The results were interpreted as positive when concentrations of specific IgE antibodies were above the manufacturer's cutoff (≥0.35 kU/L). In addition, we report our results using the established and more sensitive cutoff of 0.1 kU/L (23). All the included patients provided written informed consent before their inclusion in the study, indicating that their patient data may be used for academic research. The study was approved by the ethics committee of the University Medical Centre Goettingen (ref. 01/12/21). The study was conducted in accordance with the local legislation and institutional requirements.

Individuals who reported previous anaphylaxis caused by fish or seafood during the allergological assessment were excluded from further analyses. Statistical analyses were conducted using the *gtsummary* (v2.0.0) and *ggplot2* (v3.5.1) packages in R 4.2.1 (R Project; http://www.r-project.org). For group comparisons, we applied Wilcoxon rank sum tests. Linear regressions were performed using the Pearson method in the *ggpubr* package (v0.6.0).

## Results

### An asymptomatic elevation in the levels of sIgE against German cockroach, crab, clam, and shrimp whole allergen extract was frequently found

**Figure 1:**
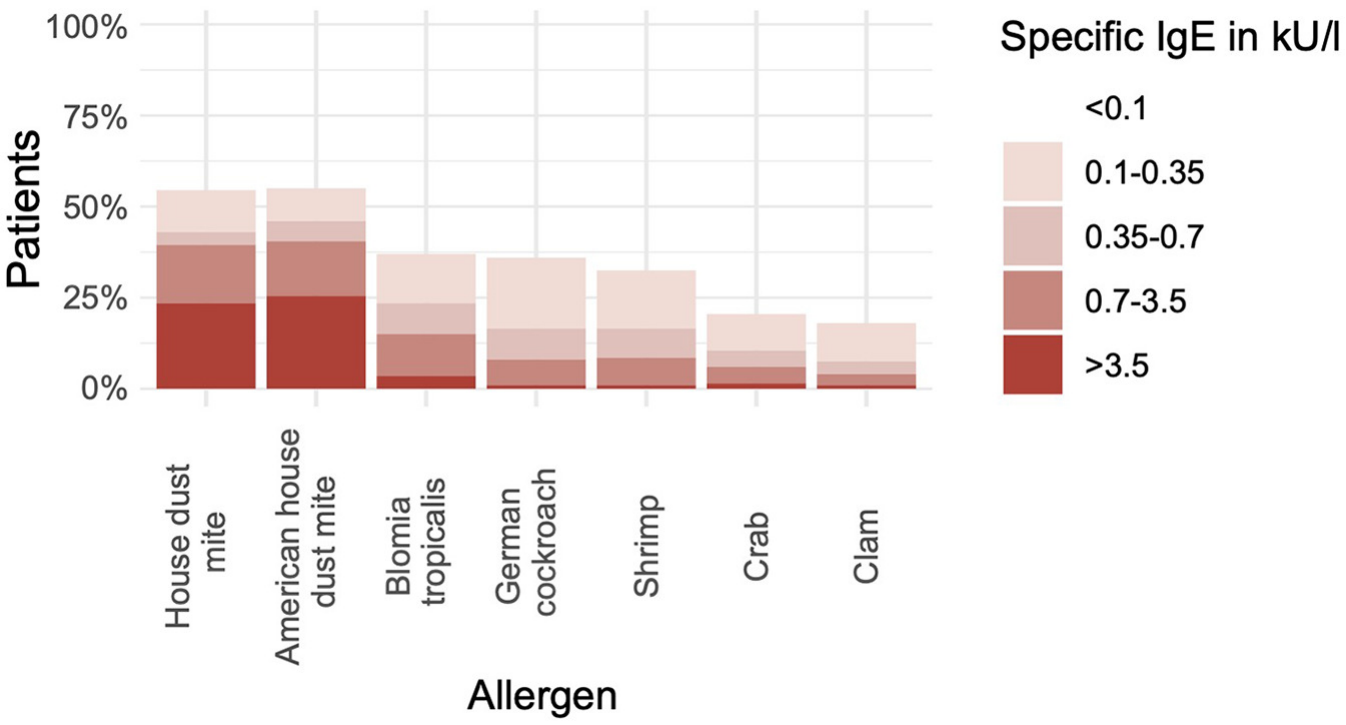
Many subjects without seafood allergy are sensitized to shellfish allergens. The bar chart shows the percentage of participants with positive results (*y*-axis) in measurements of sIgE against various allergen extracts (*x*-axis) (*n* = 198).

Of the 200 participants with elevated levels of sIgE against at least one allergen, two reported previous anaphylaxis against fish or seafood allergens and were excluded from further analyses (Supplementary Table S1). The patient characteristics are available in the Supplementary Material (Supplementary Table S2). Using the recommended cutoff of 0.35 kU/L, 86 (43%) participants were positive for sIgE against HDM, 92 (46%) for sIgE against American HDM, 47 (24%) for sIgE against *B. tropicalis*, 15 (8%) for sIgE against clam, 21 (21%) for sIgE against crab, 33 (17%) for sIgE against German cockroach, and 33 (17%) for sIgE against shrimp (Figure 1, Supplementary Table S2). Applying the more sensitive cutoff of ≥0.1 kU/L for sIgE revealed that 72 (36%) of the total population (*n* = 198) were positive for sIgE against German cockroach and 65 (33%) displayed elevated levels of anti-shrimp IgE (Figure 1).

### Sensitization to German cockroach, crab, clam, and shrimp is more common in individuals with sensitization to mites

Of the 198 patients, 93 (47%) were sensitized to mites (HDM, American HDM, and/or *B. tropicalis*), and 105 (53%) were not sensitized to mites (Table 1). Participants with mite sensitization displayed an elevated prevalence of perennial asthma or rhinitis (*p* < 0.001, Supplementary Table S2). Subjects sensitized to mites showed significantly increased levels of sIgE against clam, crab, German cockroach, and shrimp (all *p* < 0.001, Table 1).

**Table 1:** The levels of sIgE against clam, crab, German cockroach, and shrimp in subjects with sensitization to mites (HDM, American HDM, or *B. tropicalis*) (*n* = 93, “pos”) vs. those without sensitization (*n* = 105, “neg”).

Characteristic	Overall, *n* = 198	N**eg,** *n* =** 105**	Pos, *n* = 93	*p*-value^a^
Clam sIgE				<0.001
Mean (SD)	0.25 (1.27)	0.13 (0.48)	0.38 (1.78)	
Median (Q1, Q3)	0.03 (0.02, 0.07)	0.02 (0.01, 0.04)	0.03 (0.02, 0.10)	
(Min, Max)	(0.01, 13.99)	(0.01, 3.18)	(0.01, 13.99)	
Crab sIgE				<0.001
Mean (SD)	0.22 (0.83)	0.04 (0.16)	0.41 (1.16)	
Median (Q1, Q3)	0.01 (0.00, 0.08)	0.00 (0.00, 0.02)	0.06 (0.01, 0.23)	
(Min, Max)	(0.00, 7.84)	(0.00, 1.47)	(0.00, 7.84)	
German cockroach sIgE				<0.001
Mean (SD)	0.38 (1.55)	0.15 (0.41)	0.65 (2.20)	
Median (Q1, Q3)	0.06 (0.03, 0.21)	0.04 (0.03, 0.08)	0.13 (0.05, 0.36)	
(Min, Max)	(0.02, 17.32)	(0.02, 3.07)	(0.02, 17.32)	
Shrimp sIgE				<0.001
Mean (SD)	0.25 (0.68)	0.09 (0.32)	0.43 (0.90)	
Median (Q1, Q3)	0.05 (0.02, 0.14)	0.03 (0.02, 0.06)	0.11 (0.04, 0.45)	
(Min, Max)	(0.00, 5.95)	(0.00, 3.14)	(0.00, 5.95)	

Cutoff of 0.35 kU/L.

^a^ Wilcoxon rank sum test.

As expected, correlation analyses revealed strong and significant linear correlations between HDM, American HDM, and *B. tropicalis*. sIgE against HDM correlated significantly, yet weakly, with crab (R^2^ = 0.025, *p* = 0.025) and shrimp sIgE (R^2^ = 0.036, *p* = 0.0072) (Supplementary Figure S1).

Next, we analyzed all the participants without mite sensitization but with sIgE against clam, crab, German cockroach, or shrimp (cutoff of ≥0.35 kU/L). We found that in the participants without mite sensitization, asymptomatic sensitizations against clam or crab were never isolated but always coincided with German cockroach (three of four subjects) and/or shrimp sensitization (two of four subjects). Asymptomatic shrimp sensitization was found in two participants without mite or German cockroach sensitization. Subjects without sensitization to mites (HDM, American HDM, or *B. tropicalis*), but with sIgE against crab, German cockroach, clam, or shrimp, were sensitized to German cockroach in 9 of 11 cases (82%, Table 2).

**Table 2:** List of patients without sIgE against mites (incl. *B. tropicalis*) but with positive for sIgE against either clam, crab, German cockroach, or shrimp (cutoff of ≥0.35 kU/L, *n* = 11).

Patient no.	Clam	Crab	Cockroach	Shrimp
1	Pos	—	Pos	—
2	—	—	—	Pos
3	Pos	Pos	Pos	Pos
4	—	—	Pos	—
5	—	—	Pos	—
6	Pos	Pos	Pos	—
7	—	—	Pos	Pos
8	Pos	—	—	Pos
9	—	—	Pos	—
10	—	—	Pos	—
11	—	—	Pos	—

### Cross-reactivity can be explained by tropomyosin epitopes

**Figure 2:**
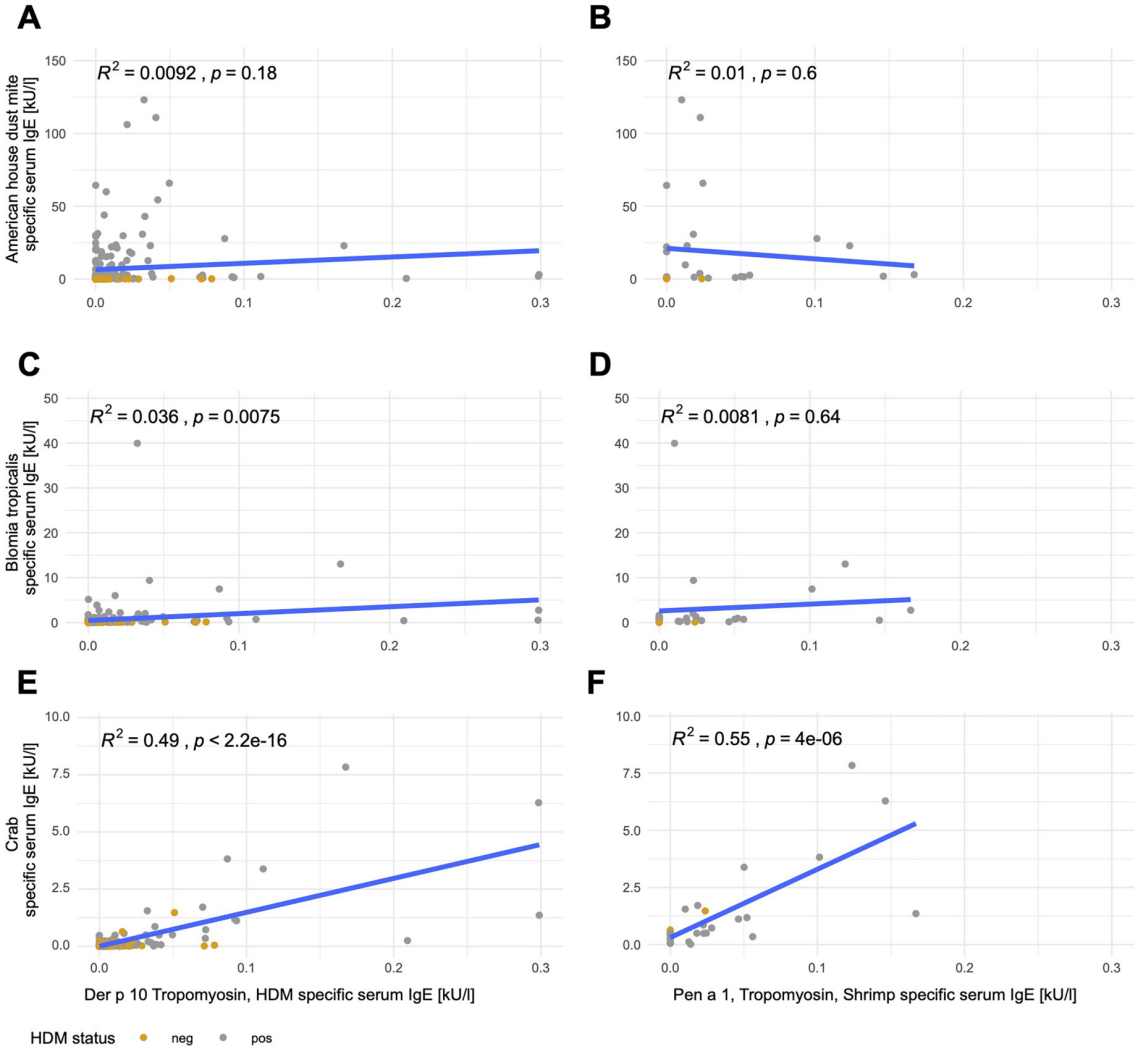
Tropomyosin sIgE correlates with crab sIgE but not mite sIgE. Linear correlations of Der p 10 (left; **A,C,E**; *n* = 198) or Pen a 1 (right; **B,D,F**; *n* = 30) with anti-American HDM **(A,B)**, anti-*B. tropicalis* **(C,D)**, and anti-crab **(E,F)** sIgE. Mite-positive subjects [sensitization to mites (HDM, American HDM, or *B. tropicalis* with a cutoff of 0.35 kU/L] are depicted in gray and mite-negative subjects are depicted in yellow.

To evaluate potential cross-reactivity, we measured anti-tropomyosin IgE antibodies against Der p 10 (HDM tropomyosin, *n* = 198) and, in a smaller subpopulation, against Pen a 1 (shrimp tropomyosin, *n* = 30). Both were highly correlated (R^2^ = 0.91, *p* < 0.001, Supplementary Figure S2). Anti-Der p 10 and Pen a 1 sIgE levels were below the cutoff of 0.35 kU/L in all subjects (Supplementary Table S3, Figure S3). The linear correlations between anti-tropomyosin IgE antibodies against both species and anti-American HDM and anti-*B. tropicalis* sIgE levels were weak. Less than 5% of anti-American HDM and anti-*B. tropicalis* sIgE was explained by the tropomyosin from the respective species (R^2^ < 5%, Figures 2A–D). In contrast, anti-crab sIgE levels were explained by approximately 50% of the same subject’s anti-tropomyosin IgE levels (R^2^ = 0.49, *p* < 0.001 for Der p 10; R^2^ = 0.55, *p* < 0.001 for Pen a 1, Figures 2E,F).

**Figure 3:**
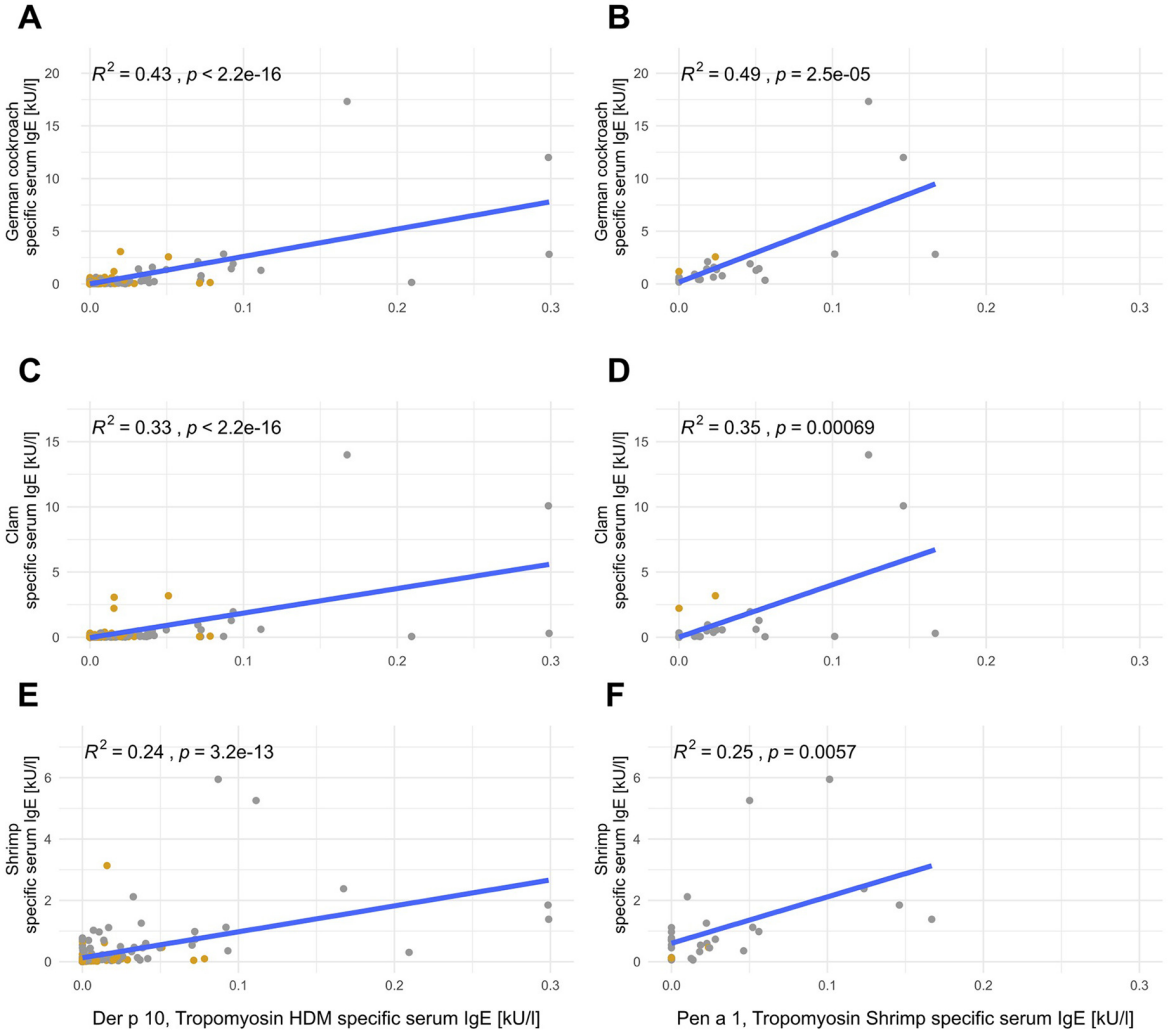
Tropomyosin sIgE correlates with German cockroach sIgE, clam sIgE, and shrimp sIgE. Linear correlations of Der p 10 (left; **A,C,E**; *n* = 198) or Pen a 1 (right; **B,D,F**; *n* = 30) with anti-German cockroach **(A,B)**, anti-clam **(C,D)**, and anti-shrimp **(E,F)** sIgE. Mite-positive subjects [sensitization to mites (HDM, American HDM, or *B. tropicalis* with a cutoff of 0.35 kU/L] are depicted in gray and mite-negative subjects are depicted in yellow.

Anti-German cockroach sIgE levels correlated with both anti-HDM and anti-shrimp tropomyosin sIgE levels, with 43% of the variation in anti-German cockroach sIgE levels explainable by anti-HDM tropomyosin sIgE levels (Der p 10, *p* < 0.001, Figure 3A) and 49% explainable by anti-shrimp tropomyosin sIgE levels (Pen a 1, *p* < 0.001, Figure 3B). Anti-Der p 10 sIgE levels explained 33% (*p* < 0.001, Figure 3C) and anti-Pen a 1 sIgE levels explained 35% of anti-clam sIgE levels (*p* < 0.001, Figure 3D). Anti-shrimp sIgE levels could be explained by anti-HDM tropomyosin sIgE levels (Der p 10) by up to 24% (*p* < 0.001, Figure 3E) and by anti-shrimp tropomyosin sIgE levels (Pen a 1) by up to 25% (*p* < 0.001, Figure 3F).

## Discussion

This study aimed to evaluate the clinical relevance of cross-reactivity between specific aeroallergens (cockroaches and mites) and food allergens (specifically, shellfish), and the potential benefit of component-resolved diagnostics. In addition, the study investigated the potential risk of false-positive results in shellfish food allergy diagnostics among subjects sensitized to mites and/or cockroaches. The study established that subjects with mite sensitization had significantly elevated sIgE levels against shellfish allergens. Intriguingly, only one isolated instance of asymptomatic sensitization to shellfish antigens occurred out of 198 cases. In all the other cases, multiple sensitizations to shellfish, cockroaches, and/or mites were observed. Thus, we conclude that sensitizations to shellfish and mites frequently coincide.

Evidence suggests that shellfish allergens exhibit significant cross-reactivity among species (24). A plethora of proteins, including tropomyosin, arginine kinase, myosin light chain, sarcoplasmic calcium-binding protein, hemocyanin, troponin C, triose phosphate isomerase, and others, are evolutionarily conserved proteins present in many species, potentially causing cross-reactivity (19). Notably, in shrimp allergy cases, tropomyosin is the most recognized allergen component represented by sIgE antibodies in individuals who test positive in food challenges (25).

Prior studies have proposed that tropomyosin acts as a cross-sensitizing pan-allergen among related species, such as crustaceans, cockroaches, and house dust mites (22). Moreover, the amino acid sequences of tropomyosin overlap by 91%–100% between shrimps, prawns, lobsters, and crabs (26). Allergenic tropomyosin is found in invertebrates such as crustaceans (shrimp, lobster, crab, and crawfish), arachnids (such as HDM), insects (such as cockroaches), and mollusks (for example, squid), while tropomyosin from vertebrates is typically non-allergenic.

In our investigation, we measured the levels of sIgE against shrimp and mite tropomyosin and discovered that both correlated significantly with the levels of sIgE against shellfish in our population of subjects who do not suffer from actual seafood allergies. This finding was corroborated by a prior study that, through immunoblot analysis, confirmed in a cohort of eight patients with a shrimp allergy that anti-shrimp-tropomyosin sIgE may also detect mite, cockroach, and lobster tropomyosin (27). Further contextualizing this link is the observation that not only do tropomyosin amino acid sequences overlap extensively among crustaceans, but they also overlap with mite tropomyosin (for example, shrimp/HDM = 81%) (22). Moreover, tropomyosin-depleted shrimp extract has been demonstrated to be considerably less effective, approximately 100 times less, in inhibiting HDM-specific IgE-binding relative to regular shrimp extract (20, 21).

Before this study, the real-world implications of tropomyosin-based cross-reactivity remained undefined. Our study revealed that tropomyosin antibodies can explain up to 55% of the variation in sIgE against specific seafoods. Among the 198 subjects tested, 33 returned positive results for shrimp sIgE according to a conservative cutoff (≥0.35 kU/L), whereas 65 tested positive when the more sensitive cutoff (≥0.1 kU/L) was used. None of these subjects had previous records of severe allergic reactions to seafood (anaphylaxis). It appears that despite significant cross-reactivity, many of the cases lacked clinical significance. In subjects sensitized to mites and/or cockroaches, it seems that a large portion of the shellfish sIgE findings could be false-positives, a result of serological cross-reactivity to aeroallergens.

Our results pose significant questions about the utility of the analysis of sIgE against shellfish allergens in patients with mite allergies. However, the reliability of our results may be constrained due to our reliance on the patients’ historical accounts to evaluate cases of anaphylaxis. Although conducting food challenges could have offered another approach, we consider it highly unlikely that subjects would forget instances of their own previous anaphylaxis or have never come into contact with shellfish. Given the notable allergenicity and cross-reactivity across different shellfish species, it seems unlikely that respective food allergies go undiagnosed. Despite OAS relating to seafood being previously reported (20), we opted not to investigate it further. Aside from the limited clinical relevance of OAS, this decision hinges on the fact that none of our study subjects reported OAS to seafood, and even following an extensive work-up, including food challenges, we did not anticipate gathering enough positive cases to permit meaningful statistical analysis.

Our findings bear clinical relevance given that HDMs are ranked among the top ten aeroallergens in Germany, with a recorded IgE seroprevalence of 15.9% in adults between 2008 and 2011 (7). Furthermore, the prevalence of mite sensitization is far higher in tropical regions (with up to 87% of allergic patients) (28). Besides peculiarities in mite allergen exposure (29), this high rate of sensitization in tropical regions may partly be attributed to cross-reactive tropomyosin antigens between mites and helminths, e.g., *Ascaris* (30).

The cross-reactivity with cockroach allergens is additionally of clinical relevance, given that arthropods and invertebrates, such as cockroaches, are common causative agents of allergic diseases in US households. In the German cockroach (*B. germanica*), Bla g 1–5 are considered major antigens, while tropomyosin (Bla g 7) is a minor antigen (31). Depending on the population and geographical area, the prevalence among cockroach-sensitized subjects for IgE against tropomyosin (Bla g 7) is approximately 18% (32).

To guarantee that the subjects included in the study were capable of mounting IgE hypersensitivity reactions, we only selected patients with at least one sIgE sensitization. To curtail selection bias, we opted to assess patients without a history of anaphylaxis. We registered a much higher frequency of sensitization against HDM (43%) compared to the general (unselected) German population (15.9%) (7). Interestingly, although there is a prevalence of sensitization against HDM (*D. pteronyssinus*) of approximately 15.9% in Germany, a mere 0.5% of German adults show IgE sensitization to shrimp tropomyosin Pen a 1 (a recombinant tropomyosin from *P. aztecus*, also known as brown shrimp) (7). Consistent with these findings, we did not register any subjects positive for sIgE against mite or shrimp tropomyosin. Conversely, other studies have reported that approximately 10% of mite-allergic individuals in Europe exhibit measurable IgE reactivity against mite tropomyosin (Der p 10) (33).

There is functional evidence of sIgE interference between tropomyosin and other epitopes, and we found a strong correlation between anti-tropomyosin sIgE and the corresponding allergen extracts. However, the scale of anti-tropomyosin sIgE antibodies (all below 0.35 kU/L) appeared insufficient to account for the serological cross-reactivity observed (mean level of sIgE against shrimp of 0.43 kU/L), at least in our cohort. It needs to be taken into account that comparing the level of sIgE against allergen extracts with that against allergen components may only be indicative. Yet, the disparity in magnitude between the sIgE results for the full allergen extracts and anti-tropomyosin molecular components remains perplexing. It would have been interesting to evaluate other molecular allergens, such as arginine kinase, however, none are commercially available for regular ImmunoCAP analyses. In addition, optimizing the composition of the full body extracts for the allergen tests may be an option to improve the specificity of positive results.

We infer from these observations that anti-tropomyosin sIgE measurements and any other currently available component-resolved diagnostics may not be helpful in estimating potential cross-reactivity at an individual level. We instead recommend a cautious interpretation of shellfish (full allergen extract) sIgE results in patients with a history of perennial aeroallergy. Further studies could consider the potential association between anti-tropomyosin sIgE and total IgE levels in asymptomatic shellfish sensitization, a relationship previously demonstrated for asymptomatic sensitization to other allergens, such as insect venoms (34).

## Data Availability

The original contributions presented in the study are included in the article/Supplementary Material, further inquiries can be directed to the corresponding author.
