# Supplementary material for: Tropomyosin-based cross-reactivity and asymptomatic shellfish sensitization in patients with perennial allergy
Source: Front Allergy. 2025 Jun 27;6:1598583. doi: 10.3389/falgy.2025.1598583 (PMC12245884; doi:10.3389/falgy.2025.1598583)
Supplement: Supplementary file 1 [file Datasheet1.docx]

**Tropomyosin-Based Cross-Reactivity and Asymptomatic Shellfish Sensitization in Patients with Perennial Allergy**

Moritz M Hollstein^1^, Marie C Schuppe^1^, Katharina K Hahn^1^, Prasad Dasari^1^, Susann Forkel^1^, Caroline Beutner^1^, Timo Buhl^1,2^

^1^Department of Dermatology, Venereology and Allergology, University Medical Centre Göttingen, Göttingen, Germany

^2^Lower Saxony Institute of Occupational Dermatology, University Medical Centre Göttingen, Göttingen, Germany

# Supplementary Figures and Tables

Table S1. Allergic comorbidity.

| **Characteristic** | **n = 200***^1^* |
| --- | --- |
| IgE against house dust mite |  |
| *neg* | 112 (56%) |
| *pos* | 88 (44%) |
| IgE against american house dust mite |  |
| *neg* | 106 (53%) |
| *pos* | 94 (47%) |
| IgE against *Blomia tropicalis* |  |
| *neg* | 151 (76%) |
| *pos* | 49 (25%) |
| Rhinitis or asthma all year |  |
| *no* | 136 (68%) |
| *yes* | 64 (32%) |
| Rhinitis or asthma during spring |  |
| *no* | 117 (59%) |
| *yes* | 83 (42%) |
| Rhinitis or asthma during summer |  |
| *no* | 135 (68%) |
| *yes* | 65 (33%) |
| Rhinitis or asthma during fall |  |
| *no* | 192 (96%) |
| *yes* | 8 (4.0%) |
| Diagnosed vespid allergy |  |
| *no* | 171 (86%) |
| *yes* | 29 (15%) |
| Diagnosed bee allergy |  |
| *no* | 192 (96%) |
| *yes* | 8 (4.0%) |
| Oral allergy syndrome triggered by fruits or vegetables |  |
| *no* | 152 (76%) |
| *yes* | 48 (24%) |
| Oral allergy syndrome triggered by nuts |  |
| *no* | 168 (84%) |
| *yes* | 32 (16%) |
| Oral allergy syndrome triggered by meat |  |
| *no* | 198 (99%) |
| *yes* | 2 (1.0%) |
| Oral allergy syndrome triggered by milk |  |
| *no* | 200 (100%) |
| Oral allergy syndrome triggered by fish or seafood |  |
| *no* | 200 (100%) |
| Oral allergy syndrome triggered by cereals |  |
| *no* | 200 (100%) |
| Anaphylaxis because of fruits or vegetables |  |
| *no* | 191 (96%) |
| *yes* | 9 (4.5%) |
| Anaphylaxis because of nuts |  |
| *no* | 188 (94%) |
| *yes* | 12 (6.0%) |
| Anaphylaxis because of meat |  |
| *no* | 200 (100%) |
| Anaphylaxis because of milk |  |
| *no* | 199 (100%) |
| *yes* | 1 (0.5%) |
| Anaphylaxis because of fish or seafood |  |
| *no* | 198 (99%) |
| *yes* | 2 (1.0%) |
| Anaphylaxis because of cereals |  |
| *no* | 199 (100%) |
| *yes* | 1 (0.5%) |
| *^1^*n (%), cutoff 0.35 kU/l. | |

Table S2:

| **Characteristic** | **Overall** n = 198*^1^* | **neg** n = 105*^1^* | **pos** n = 93*^1^* |
| --- | --- | --- | --- |
| Sex |  |  |  |
| *female* | 150 (76%) | 78 (74%) | 72 (77%) |
| *male* | 48 (24%) | 27 (26%) | 21 (23%) |
| Age |  |  |  |
| *10-19* | 10 (5.1%) | 2 (1.9%) | 8 (8.6%) |
| *20-29* | 45 (23%) | 14 (13%) | 31 (33%) |
| *30-39* | 38 (19%) | 22 (21%) | 16 (17%) |
| *40-49* | 35 (18%) | 20 (19%) | 15 (16%) |
| *50-59* | 35 (18%) | 22 (21%) | 13 (14%) |
| *60-69* | 24 (12%) | 17 (16%) | 7 (7.5%) |
| *70-79* | 11 (5.6%) | 8 (7.6%) | 3 (3.2%) |
| House dust mite |  |  |  |
| *neg* | 112 (57%) | 105 (100%) | 7 (7.5%) |
| *pos* | 86 (43%) | 0 (0%) | 86 (92%) |
| American house dust mite |  |  |  |
| *neg* | 106 (54%) | 105 (100%) | 1 (1.1%) |
| *pos* | 92 (46%) | 0 (0%) | 92 (99%) |
| *Blomia tropicalis* |  |  |  |
| *neg* | 151 (76%) | 105 (100%) | 46 (49%) |
| *pos* | 47 (24%) | 0 (0%) | 47 (51%) |
| Crab |  |  |  |
| *neg* | 177 (89%) | 103 (98%) | 74 (80%) |
| *pos* | 21 (11%) | 2 (1.9%) | 19 (20%) |
| German Cockroach |  |  |  |
| *neg* | 165 (83%) | 96 (91%) | 69 (74%) |
| *pos* | 33 (17%) | 9 (8.6%) | 24 (26%) |
| Clam |  |  |  |
| *neg* | 183 (92%) | 101 (96%) | 82 (88%) |
| *pos* | 15 (7.6%) | 4 (3.8%) | 11 (12%) |
| Shrimp |  |  |  |
| *neg* | 165 (83%) | 101 (96%) | 64 (69%) |
| *pos* | 33 (17%) | 4 (3.8%) | 29 (31%) |
| Rhinitis or asthma all year^2^ |  |  |  |
| *no* | 136 (69%) | 85 (81%) | 51 (55%) |
| *yes* | 62 (31%) | 20 (19%) | 42 (45%) |
| *^1^*n (%).^2^ Pearson’s Chi-squared test: p < 0.001, cutoff 0.35 kU/l. | | | |

Table S3:

| **Characteristic** | **Overall** n = 198*^1^* | **neg** n = 105*^1^* | **pos** n = 93*^1^* |
| --- | --- | --- | --- |
| Der p 10 Tropomyosin, HDM |  |  |  |
| *neg* | 198 (100%) | 105 (100%) | 93 (100%) |
| *pos* | 0 (0%) | 0 (0%) | 0 (0%) |
| Pen a 1, Tropomyosin, Shrimp |  |  |  |
| *neg* | 29 (100%) | 2 (100%) | 27 (100%) |
| *pos* | 0 (0%) | 0 (0%) | 0 (0%) |
| *(Missing)* | 169 | 103 | 66 |
| *^1^*n (%), 0.35 kU/l, cutoff 0.35 kU/l. | | | |

Supplementary Figures

Figure S1: **HDM-specific sIgE correlates with other mite allergens and some shellfish whole extract sIgE.** A-D: Houst dust mite vs other allergens. Red lines indicate sIgE cutoffs (0.35 kU/l), n=198.

Figure S2: **Anti-shrimp and anti-HDM tropomyosin sIgE is strongly correlated.** Correlation between sIgE against tropomyosin from house dust mites (HDM, Der p 10, y-axis) and shrimps (Pen a 1, x-axis) (subgroup, n=30).

Figure S3: **All tropomyosin sIgE measurements were below 0.35 kUA/l.** Percentage of participants with positive results (y-axis) in measurements of sIgE against Der p 10 (HDM tropomyosin, n=198)) and Pen a 1 (shrimp tropomyosin, n=30) among study patients.
